# Associations of MC1R Substitutions With Body Color Variation in a Desert Lizard

**DOI:** 10.1002/ece3.73481

**Published:** 2026-04-23

**Authors:** Haojie Tong, Qianjin Lin, Shuangyue Shi, Kaixin Wang, Yaxin Guo, Richard P. Brown, Yuanting Jin

**Affiliations:** ^1^ College of Life Sciences China Jiliang University Hangzhou P. R. China; ^2^ School of Biological and Environmental Sciences Liverpool John Moores University Liverpool UK

**Keywords:** binding affinity, body coloration, MC1R, melanistic

## Abstract

Body color variation is widespread among animals, and the melanocortin 1 receptor (MC1R) acts as a vital switch gene in regulating pigmentation. A small number of amino acid substitutions, or even a single one, in MC1R are known to influence the body coloration of animals. To investigate the associations between MC1R variants and body color variation in melanistic and non‐melanistic populations of a toad‐headed lizard, *P. versicolor* , we obtained the complete coding sequences of the *MC1R* gene. Sequence analysis identified two amino acid substitutions—V165I and V237I, located in transmembrane domains 4 (TM4) and 6 (TM6), respectively—that were significantly associated with color variation in *P. versicolor* . In silico structural analysis incorporating these two substitutions demonstrated that the melanistic MC1R exhibited stronger binding affinity for α‐MSH, as characterized by a smaller binding surface area and volume, a greater number of hydrogen bonds, more favorable binding free energy, a higher binding constant, and a lower dissociation constant. These changes may ultimately enhance melanin synthesis in melanistic populations. Our results provide insights into how amino acid substitutions affect receptor–ligand‐binding capacity by altering the pocket size of MC1R in *P. versicolor* .

## Graphical abstract

We successfully identified two amino acid substitutions (V165I and V237I) in MC1R that are significantly associated with body color variation between melanistic and non‐melanistic populations of a toad‐headed lizard, *P. versicolor* . In silico analysis revealed that both substitutions located in transmembrane (TM) domains affect the binding affinity between α‐MSH and MC1R. Specifically, these substitutions reshape the size of the binding pocket, which may ultimately lead to distinct body coloration in this species.

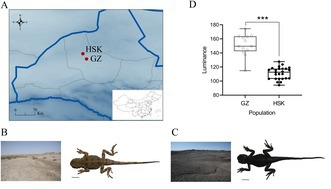

## 1 Introduction

Body color variation is widespread among animals, and it is primarily regulated by the pigment contents produced by melanocytes. In these cells, the melanocortin 1 receptor (MC1R) gene plays a pivotal role in melanin synthesis. Structurally, its encoded MC1R protein is a G protein‐coupled receptor (GPCR) expressed on the surface of melanocytes, consisting of seven transmembrane (TM) α‐helical domains, three extracellular loops (ELs), three intracellular loops (ILs), an extracellular N‐terminus, and an intracellular C‐terminus (Horrell et al. 2016). Activation of MC1R by alpha‐melanocyte‐stimulating hormone (α‐MSH, a 13‐residue peptide) can induce the production of cyclic adenosine monophosphate (cAMP) and promote melanin synthesis, resulting in black pigmentation (Garcia‐Borron et al. 2005; Horrell et al. 2016). In contrast, lighter body colors (such as red or yellow) occur when MC1R is inhibited by agouti‐signaling protein (Barsh et al. 2000). Therefore, MC1R acts as a key switch in these pigment synthesis processes. Alterations in its expression level or genetic variants are believed to influence coloration across a wide range of vertebrate species, including fishes (Richardson et al. 2008; Wang et al. 2020; Song et al. 2022), amphibians (Matsuba 2012), reptiles (Rosenblum et al. 2004; Nunes et al. 2011; Laurent et al. 2016; Jin et al. 2020; Tong et al. 2023; Garcia‐Elfring et al. 2025), birds (Schwochow et al. 2021; Qi et al. 2023), and mammals (Valverde et al. 1995; Nachman et al. 2003; Xiong et al. 2016; Kawaguchi et al. 2025).

Reptiles tend to match their body coloration to their living substrate for camouflage, and the MC1R is one of the most important genes regulating this adaptation. Although several studies have failed to detect a statistical association between MC1R variants and color variation (Corso et al. 2012; Buades et al. 2013; Cox et al. 2013), a total of 11 amino acid substitutions (Table 1) have so far been identified as related to such color variation in reptiles, including *Lacerta lepida lepida* (Nunes et al. 2011), three White Sands lizard species (Rosenblum et al. 2004), two *Phrynocephalus* lizard species (Jin et al. 2020; Tong et al. 2023), and *Python regius* (Garcia‐Elfring et al. 2025). Furthermore, some studies have explored the molecular and functional mechanisms underlying the detected MC1R substitutions. Specifically, the T170I substitution in *Aspidoscelis inornata* impairs receptor signaling, while the H208Y substitution in *S. undulatus* (Rosenblum et al. 2010) and the E183K substitution in *P. erythrurus* (Tong et al. 2023) affect the integration of the receptor into the melanophore membrane—all of these changes lead to alterations in melanin production. Therefore, consistent with the well‐characterized mechanism in mammals (Ringholm et al. 2004), independent MC1R substitutions can drive melanin‐based color variation in reptiles.

**TABLE 1:** Association between variants and body colors in *P. versicolor* .

Variant	Amino acid substitution	Genotype frequency						*p*
		Non‐melanistic			Melanistic			
C51T	—	13(CC) (0.76)	1(CT) (0.06)	3(TT) (0.18)	27(CC) (0.52)	12(CT) (0.23)	13(TT) (0.25)	—
C483T	—	13(CC) (0.76)	3(CT) (0.18)	1(TT) (0.06)	31(CC) (0.60)	11(CT) (0.21)	10(TT) (0.19)	—
G493A	V165I	17(GG) (1.00)	0(GA) (0.00)	0(AA) (0.00)	42(GG) (0.81)	2(GA) (0.04)	8(AA) (0.15)	0.007*
A504G	I168M	3(AA) (0.18)	7(AG) (0.41)	7(GG) (0.41)	15(AA) (0.29)	13(AG) (0.25)	24(GG) (0.46)	0.842
C540T	—	8(CC) (0.47)	6(CT) (0.35)	3(TT) (0.18)	45(CC) (0.86)	6(CT) (0.12)	1(TT) (0.02)	—
C552T	—	9(CC) (0.53)	5(CT) (0.29)	3(TT) (0.18)	44(CC) (0.85)	7(CT) (0.13)	1(TT) (0.02)	—
T600C	—	17(TT) (1.00)	0(TC) (0.00)	0(CC) (0.00)	33(TT) (0.64)	11(TC) (0.21)	8(CC) (0.15)	—
G709A	V237I	17(GG) (1.00)	0(GA) (0.00)	0(AA) (0.00)	40(GG) (0.77)	9(GA) (0.17)	3(AA) (0.06)	0.022*
C729T	—	9(CC) (0.53)	5(CT) (0.29)	3(TT) (0.18)	43(CC) (0.83)	7(CT) (0.13)	2(TT) (0.04)	—
G861A	—	17(GG) (1.00)	0(GA) (0.00)	0(AA) (0.00)	41(GG) (0.79)	9(GA) (0.17)	2(AA) (0.04)	—

* Substitutions with significant association with color variation (Fisher's exact test, *p* < 0.05).

**FIGURE 1:**
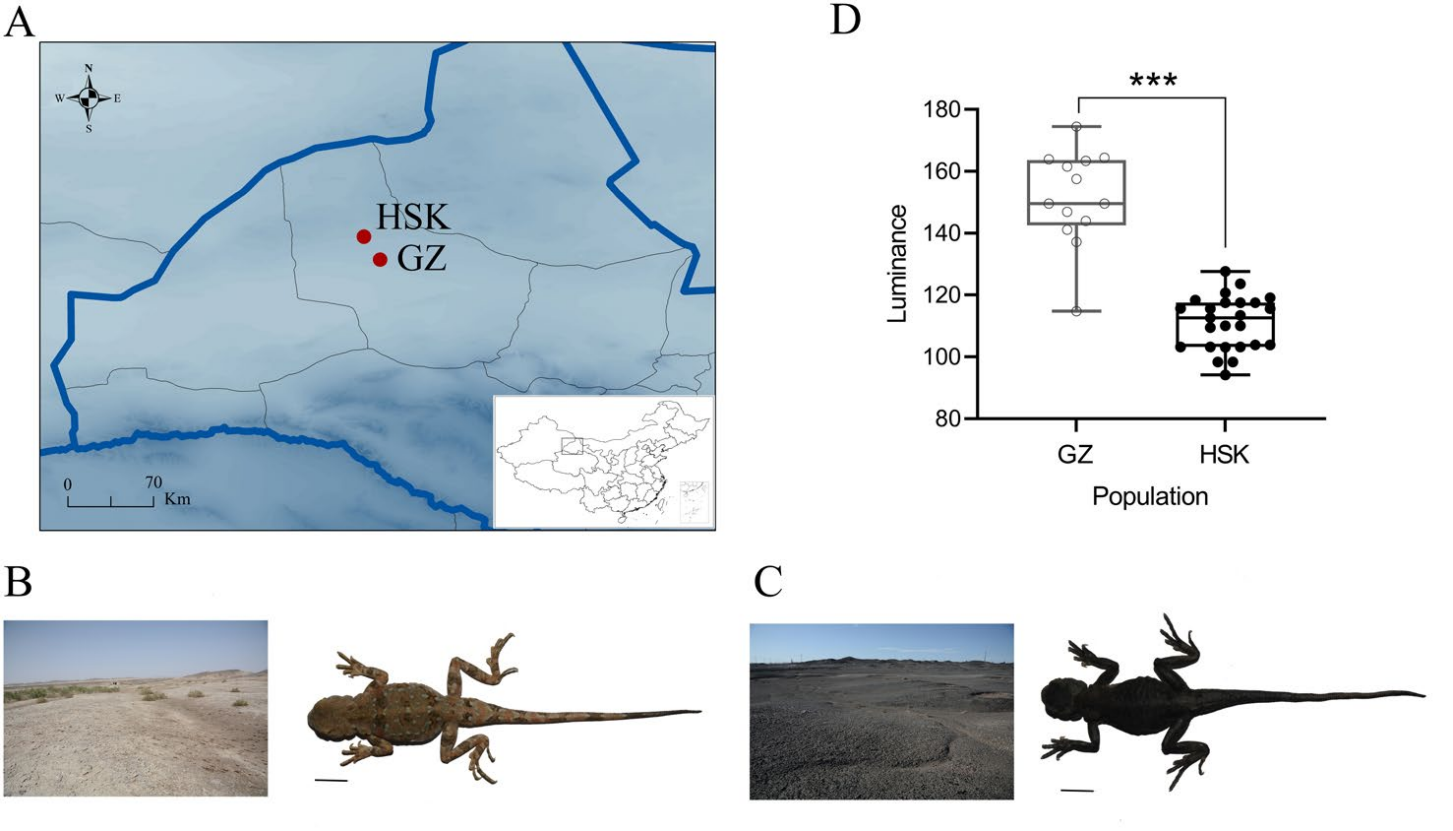
Sampling locations, substrate characteristics, and corresponding body coloration of *P. versicolor* individuals. (A) Geographic map depicting the two sampling sites (red points) in Gansu Province, China: Heishan Kou (HSK) in Liuyuan town and Guazhou (GZ) city. Blue lines represent the border of Gansu Province. (B) Non‐melanistic *P. versicolor* individual collected from GZ, where the substrate exhibits a non‐melanistic (weathered yellow) coloration. (C) Melanistic *P. versicolor* individual sampled from HSK, an area characterized by black (melanistic) substrate. Scale bar (black lines), 10 mm. (D) Luminance variation on lizard's dorsal skin between GZ and HSK populations. ***p < 0.001.

In *Phrynocephalus*, the effects of substrate color on lizards' body color variation have received considerable attention (Tao et al. 2018; Sun et al. 2024; Chen et al. 2025). One representative species of this genus is *P. versicolor* , which is widely distributed in the deserts and semideserts spanning from Ningxia to Xinjiang in China (Wang and Fu 2004) and exhibits variegated coloration, as its name implies. The population inhabiting Heishan Kou (HSK)—where the substrate is black—displays extremely melanistic body coloration (Figure 1), whereas populations from areas with weathered yellow substrate are non‐melanistic. Our previous reciprocal substrate translocation experiments have confirmed that this color variation is a morphological adaptation rather than a product of phenotypic plasticity (Tong et al. 2016, 2019). Recently, through population genetic analysis, selective sweep analysis, and differential expression analysis, we identified several genes that may be responsible for the differential pigmentation adaptation of *P. versicolor* to substrate color (Jin et al. 2022). Different genes exhibit varied functions in melanin synthesis and body color variation. Moreover, two factors—low‐coverage sequencing of individual samples and a relatively high level of genetic differentiation among populations (though *F*_st_ values were less than 0.2)—have limited the statistical power to identify key candidate genes associated with color variation in this species, such as the *MC1R* gene.

Therefore, considering the potential role of the MC1R in regulating pigmentation—even a single amino acid substitution in *P. erythrurus* can affect this process (Tong et al. 2023)—we hypothesize that MC1R may perform similar functions in *P. versicolor* . To verify this hypothesis, we designed the following research objectives: (1) obtain the complete coding sequences of the *MC1R* gene from both melanistic and non‐melanistic *P. versicolor* populations, (2) assess the potential association between *MC1R* genetic variants and body coloration, and (3) analyze the structural and functional differences of MC1R between melanistic and non‐melanistic individuals through in silico approaches.

## 2 Materials and Methods

### 2.1 Sampling Collection and Preparation

A total of 69 adult *P. versicolor* were collected in May 2017, including 52 melanistic individuals (15 males, 37 females) from HSK (96.17° E, 41.08° N, 1235 m above sea level) in Liuyuan town and 17 non‐melanistic individuals (seven males, nine females) from Guazhou (GZ; 95.61° E, 41.05° N, 1386 m above sea level), Gansu Province (Figure 1). Tail tip tissue samples were collected from each lizard and preserved in 100% ethanol. This fieldwork and sampling protocol was approved by the Animal Experiments Ethics Committee of China Jiliang University.

### 2.2 Color Measurements

Of the 69 adult *P. versicolor* individuals examined, we randomly selected 13 lizards from the GZ population (seven males, six females) and 25 from the HSK population (10 males, 15 females) for dorsal color measurements. All lizards were photographed using a digital camera (Nikon D7100), and luminance values were extracted for each individual to quantify color variation. Detailed protocols for color data collection and analysis are available in Tong et al. (2023). We used *t*‐tests to compare luminance differences between the GZ and HSK populations.

### 2.3 MC1R Amplification and Sequence Analysis

Genomic DNA was extracted from each tissue sample using the Qiagen DNeasy Blood and Tissue Kit (Qiagen, Canada) following the manufacturer's instructions. PCR amplification of the full coding sequence (CDS) of the MC1R gene was performed according to the protocols described previously (Jin et al. 2020). The PCR products were purified and subjected to Sanger sequencing by a commercial service provider. Sequence alignment, variant identification, and nucleotide‐to‐amino acid translation were conducted using MEGA v6.0 software (Tamura et al. 2013). DnaSP v. 6.12.03 (Rozas et al. 2017) was employed for sequence phasing and haplotype identification. Genotype frequencies were calculated for both the melanistic and non‐melanistic populations. For each non‐synonymous substitution, Fisher's exact test by IBM SPSS Statistics R27.1.1.0 was further used to analyze the contingency of amino acid frequencies between these two populations.

### 2.4 In Silico MC1R Structural Analysis

To explore the three‐dimensional (3D) structure of MC1R, the full‐length amino acid sequences of two individuals—GZ‐01 (female, from the non‐melanistic population) and HSK‐24 (female, from the melanistic population)—were selected for in silico structural analysis. 3D structure models for both MC1R proteins and the ligand α‐MSH (CID: 16133793, retrieved from the PubChem database) were predicted using Deep learning‐based Iterative Threading ASSEmbly Refinement (D‐I‐TASSER) with default parameters (Zheng et al. 2025). The estimated TM‐score (eTM‐score) of the 3D structures was calculated based on four criteria: the significance of threading template alignments, the contact map satisfaction rate, the mean absolute error between the model distance and the AttentionPotential distance, and the convergence of D‐I‐TASSER simulations. The eTM‐score typically ranges from 0 to 1, with higher values indicating greater model confidence (Zheng et al. 2025).

### 2.5 In Silico MC1R–α‐MSH‐Binding Analysis

Molecular docking was performed to evaluate the binding affinity between α‐MSH and each of the non‐melanistic/melanistic MC1R. Briefly, AutoDockTools v1.5.7 (Forli et al. 2016) was used for preprocessing protein molecules and small‐molecule ligands, including removing water molecules and adding hydrogen atoms. The binding pocket, pocket volume, and surface area of MC1R were predicted using ProteinsPlus (https://proteins.plus/). QuickVina‐W software (Hassan et al. 2017) was employed for molecular docking between α‐MSH and MC1R with default parameters, and the docking model with the optimal binding energy was selected for subsequent analysis. The 3D structures and docking results were visualized using PyMOL‐3.1.6 (Schrödinger and DeLano 2020).

## 3 Results

### 3.1 Variations on Dorsal Color

We found that lizards from the GZ population exhibited significantly higher dorsal luminance (151.41 ± 4.29) than those from the HSK population (111.03 ± 1.73) (*t* = 10.367, df = 36, *p* < 0.001; Figure 1D). This result indicates that the HSK population has darker dorsal coloration compared with the GZ population.

### 3.2 Genetic Variation of MC1R

The complete coding sequence (CDS) of the *MC1R* gene in *P. versicolor* is 942 bp in length. We successfully cloned the full‐length *MC1R* CDS from all 69 individuals sampled, including both melanistic and non‐melanistic populations. Sequence alignment revealed 932 conserved sites (accounting for 98.94% of the total sequence) and 10 variable sites (1.06%). All 10 variable sites were heterozygous and parsimony‐informative across all individuals. Among these, seven were synonymous substitutions, while three sites (#493, #504, and #709) resulted in non‐synonymous substitutions (Table 1, Data S1).

### 3.3 Associations Between MC1R Variants and Body Coloration

**FIGURE 2:**
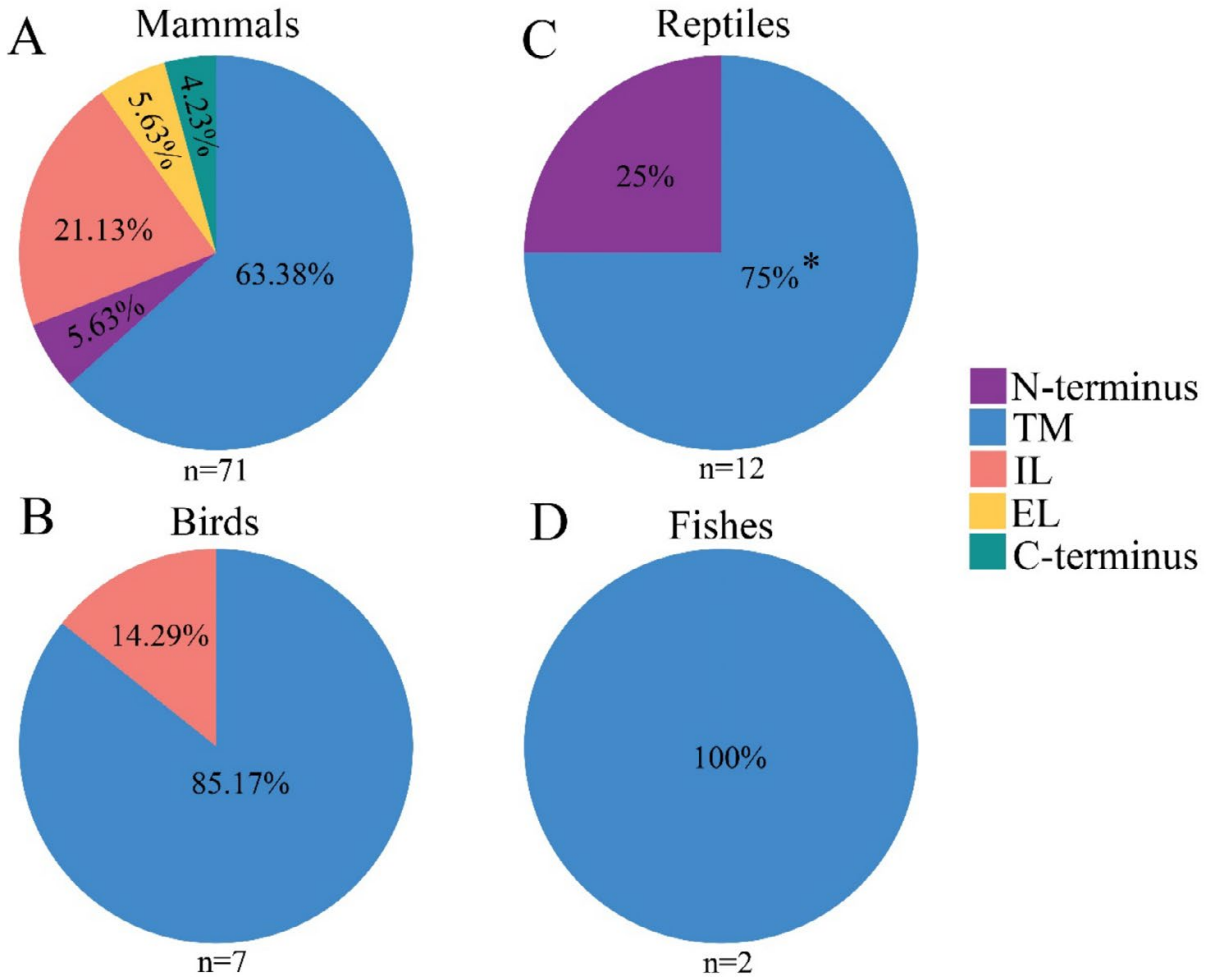
Proportions of pigmentation‐related MC1R substitutions across different structural regions of the MC1R protein. Detailed amino acid substitutions in Mammalia (A), Aves (B), Reptilia (C), and Fish (D) are provided in Table S1. The asterisk in the Reptilia panel (C) indicates the structural region corresponding to the two amino acid substitutions identified in *P. versicolor* . EL, extracellular loop; IL, intracellular loop; TM, transmembrane.

**FIGURE 3:**
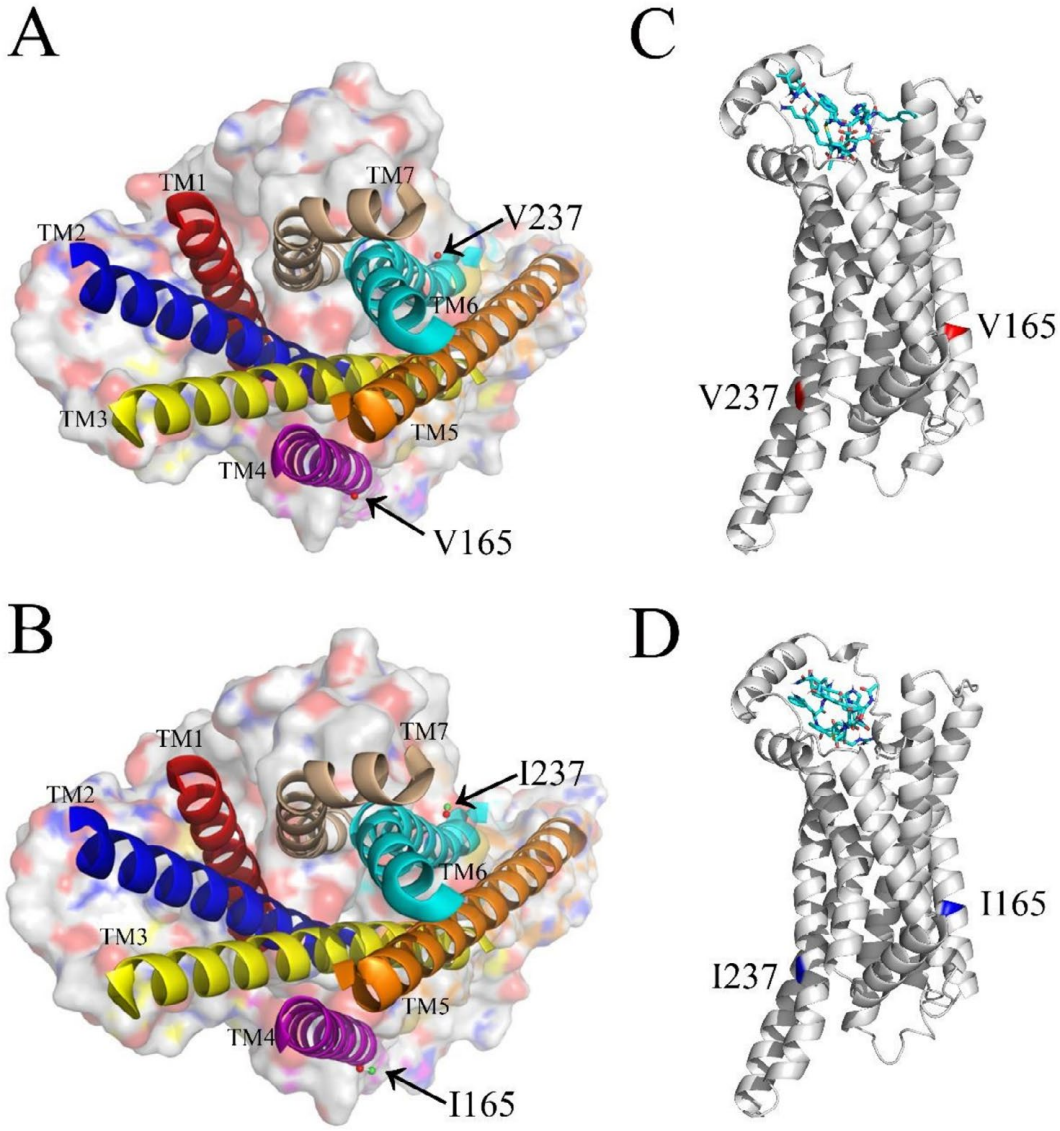
In silico predicted structures of MC1Rs from *P. versicolor* and highlights the key amino acid substitutions and binding‐related structural features. (A, B) Periplasmic (top) views of the non‐melanistic (A) and melanistic (B) MC1R structures, displayed in surface representation. The seven TM α helices are visualized as ribbons. The V165I substitution (localized in TM4) and V237I substitution (localized in TM6)—the two variants associated with color variation—are marked by green spheres, which represent the additional methyl groups introduced by these substitutions. (C, D) Ribbon representations of the non‐melanistic (C) and melanistic (D) MC1R models, viewed from within the cell membrane. Residues at positions 165 and 237 are situated in the middle and lower segments of the respective α‐helices.

Five types of MC1R protein were identified, which were characterized by three amino acid substitutions: V165I, I168M, and V237I (Table 1, File S1). Fisher's exact test showed no significant difference in the frequency of the I168M residue between melanistic and non‐melanistic populations (*p* = 0.842). In contrast, the other two residues (V165I and V237I) were significantly associated with body coloration (Fisher's exact test, *p* < 0.05) (Table 1). From the perspective of secondary structure, all three residues are located in the TM region (Figure 2), with V165I and I168M located in the TM4, while V237I resides in the TM6 (Figure 3).

### 3.4 Structural Variations of MC1R

We successfully predicted the 3D protein structures of MC1R from both melanistic and non‐melanistic individuals, which carry the two key substitutions (V165I and V237I). The eTM‐scores of the optimal models were 0.81 (melanistic) and 0.80 (non‐melanistic), respectively, indicating high reliability of the structural predictions. The periplasmic (top) view of the MC1R 3D structure (Figure 3A,B) showed that the two amino acid substitutions are localized near the bottom of the TM helices. Both the V165I and V237I substitutions introduce an additional methyl group in the amino acid side chain.

### 3.5 Binding Affinity Between α‐MSH and MC1R

The modeled MC1R–α‐MSH complex revealed a binding pocket surrounded by the seven TM helices of MC1R (Figure 3C,D). Compared with the binding pocket of MC1R from non‐melanistic individuals, the pocket in melanistic MC1R exhibited a smaller volume (1973.91 Å^3^ vs. 1705.85 Å^3^) and surface area (2223.93 Å^2^ vs. 1819.34 Å^2^) (Table 2). Molecular docking analysis further demonstrated that the melanistic MC1R–α‐MSH interaction had superior binding characteristics compared with the non‐melanistic complex: it formed more hydrogen bonds (15 vs. 12) and had a more favorable binding free energy (−7.8 kcal/mol vs. −6.7 kcal/mol), a higher binding constant (*K*_a_, 2.0 × 10^5^ vs. 1.3 × 10^4^), and a lower dissociation constant (*K*_d_, 5.0 × 10^−6^ vs. 7.7 × 10^−5^) (Table 2). Collectively, these results indicate a stronger binding affinity between MC1R and α‐MSH in melanistic *P. versicolor* relative to non‐melanistic individuals.

**TABLE 2:** The interactive parameters of α‐MSH with melanistic MC1R and non‐melanistic MC1R.

Interactive parameters	Non‐melanistic	Melanistic
Pocket surface area (Å^2^)	2223.93	1819.34
Pocket volume (Å^3^)	1973.91	1705.85
Hydrogen bonds in pocket	12	15
Δ*G* (kcal/mol)	−6.70	−7.80
*K*_a_ (M^−1^)	1.30 × 10^4^	2.00 × 10^5^
*K*_d_ (M)	7.69 × 10^−5^	5.00 × 10^−6^

## 4 Discussion

Lizards of the genus *Phrynocephalus* exhibit diverse body coloration, which enables them to adapt to variable environments—particularly through background‐matching camouflage (Tong et al. 2019; Sun et al. 2024). The melanistic population of *P. versicolor* from HSK, where the substrate is black, displays the darkest body coloration within this genus, making it an ideal model for investigating the molecular mechanisms underlying melanism in reptilian body coloration. In the present study, we successfully cloned the *MC1R* gene from both melanistic and non‐melanistic *P. versicolor* populations. Through genetic association analysis, we identified two amino acid substitutions (V165I and V237I) that were significantly correlated with color variation. In silico structural and binding analyses further confirmed that the melanistic MC1R allele exhibits stronger binding affinity to its ligand, α‐MSH. This enhanced MC1R–α‐MSH interaction is likely to promote melanin synthesis, ultimately leading to the melanistic phenotype observed in the HSK population. There is likely a relationship between MC1R and color variation in *P. versicolor* that aligns with our initial hypothesis. Notably, this gene was not detected in our previous genomic analysis (Jin et al. 2022), which suggests a critical limitation of genomic selective sweep analyses: they may fail to identify genes with important functional roles but relatively weak statistical signals of variation, especially when low‐coverage sequencing data are used. This observation highlights the value of targeted candidate gene approaches in complementing genome‐wide studies, particularly for uncovering genes involved in adaptive phenotypic traits.

The MC1R gene is well‐documented to exhibit high polymorphism across vertebrates, with at least 92 amino acid substitutions reported in this taxonomic group (Figure 2, Table S1). For lizards of the genus *Phrynocephalus*, the full‐length coding sequence (CDS) of *MC1R* (942 bp) is relatively conserved; however, 35 nuclear variable sites have been identified across the genus to date (Jin et al. 2020; Tong et al. 2023). In the present study, we detected 10 variable sites in *P. versicolor* populations (Table 1 and File S1). When comparing this variation with that of two congeneric species, the number of variable sites in *P. versicolor* is lower than that in *P. theobaldi* but higher than that in *P. erythrurus* . Among the variable sites identified in *P. versicolor* , four (C483T, G493A, C729T, and G861A) are shared with *P. theobaldi* , while only one variant (G861A) is common across all three species. These findings highlight that the *MC1R* gene exhibits varying degrees of genetic differentiation among different *Phrynocephalus* species. Given its consistent polymorphism and interspecific variation, *MC1R* could serve as a valuable candidate nuclear marker for investigating genetic diversity within the *Phrynocephalus* genus, similar to its utility in other reptilian groups (Metallinou et al. 2015; Ribeiro Jr. et al. 2022).

Of the 10 variable sites identified in *P. versicolor* in this study, three are non‐synonymous, corresponding to three amino acid substitutions: V165I, I168M, and V237I. Statistical analysis revealed that only V165I and V237I were significantly associated with color variation in this species. Notably, both functionally relevant substitutions are localized in the TM domains of the MC1R protein (Figure 3)—a region known to exhibit high mutagenicity across vertebrates, with 66.3% of all reported MC1R substitutions occurring within TM domains (File S1). However, the distribution of MC1R substitutions in *P. versicolor* differs from that in congeneric species. In *P. theobaldi* , three substitutions are located in extracellular regions and two in TM domains (Jin et al. 2020), while *P. erythrurus* harbors a single substitution exclusively in an extracellular region (Tong et al. 2023). Given that the TM domains ELs, ILs, extracellular N‐terminus, and intracellular C‐terminus of the MC1R protein each perform distinct functional roles (Guernsey et al. 2013; Hauser et al. 2022; Cavatao et al. 2024), we hypothesize that the V165I and V237I substitutions in *P. versicolor* may mediate a unique functional mechanism of MC1R regulation compared with its congenerics. More importantly, substitutions at sites adjacent to V165I have been documented in other lizard species: V168I in *Heliobolus maculatus* (Rosenblum et al. 2004) and T162I in *Lacerta lepida lepida* (Nunes et al. 2011). Intriguingly, the V168I substitution in *H. maculatus* is suspected to affect MC1R function through a distinct pathway—it neither impairs receptor signaling nor disrupts receptor integration into the melanophore membrane (Rosenblum et al. 2010). Given the proximity of these substitutions to V165I in *P. versicolor* , it is highly plausible that the V165I substitution in our study exerts its effects on melanin synthesis via a similar alternative mechanism, which warrants further experimental validation.

It is well‐documented that the seven TM domains of MCRs form the core of the ligand‐binding pocket, and substitutions within this region can directly alter the pocket structure (Yang et al. 2013; Feng et al. 2025). For instance, in mammals, substitutions such as E94A in TM2, D117A and D121A in TM3, and H260A in TM6 have been shown to modify MCR‐binding pocket properties (Frandberg et al. 1994; Yang et al. 2013). Building on this knowledge, we predicted the binding pocket structures of both non‐melanistic and melanistic MC1R in *P. versicolor* . The predicted surface areas of the binding pockets were 2223.93 Å^2^ (non‐melanistic) and 1819.34 Å^2^ (melanistic), respectively. Notably, these values are consistent with the size of the orthosteric peptide‐binding pocket of MC1R observed via cryo‐electron microscopy (cryo‐EM), which was reported to be 2085 Å^2^ (Ma et al. 2021), providing strong support for the reliability of our in silico predictions. Compared with the non‐melanistic MC1R, the melanistic variant exhibited a significantly smaller binding pocket volume and surface area. This structural difference can be attributed to the V165I and V237I substitutions localized in the TM domains; specifically, these substitutions introduce two additional methyl groups, which represent the sole chemical distinction between the melanistic and non‐melanistic MC1R isoforms (Figure 3). Therefore, these findings demonstrate a close association between MC1R amino acid mutations and binding pocket structural variation in *P. versicolor* , highlighting how targeted TM domain substitutions modulate the receptor's ligand‐binding architecture.

Hydrogen bond formation is a critical determinant of the binding interaction between the MC1R and its ligand, α‐MSH (Horrell et al. 2016; Chen et al. 2017; Ma et al. 2021). In the present study, molecular docking analysis revealed that the melanistic MC1R variant of *P. versicolor* forms a greater number of hydrogen bonds with α‐MSH compared with the non‐melanistic variant. This increase in hydrogen bonding is a key structural feature that enhances the binding capacity between the receptor and its ligand. The enhanced MC1R–α‐MSH‐binding ability of the melanistic variant is further supported by data on binding free energy, binding constants (*K*_a_), and dissociation constants (*K*_d_). The melanistic MC1R–α‐MSH complex exhibits more favorable binding free energy and high changes in K values, with the binding and dissociation constants differing by nearly one order of magnitude between the two variants (Table 2). These thermodynamic and kinetic parameters collectively confirm that the melanistic MC1R has a stronger binding affinity for α‐MSH than its non‐melanistic counterpart. Notably, this elevated binding affinity is highly likely associated with the reduced binding pocket size of the melanistic MC1R, as discussed in the previous section. The smaller pocket volume and surface area—driven by the V165I and V237I substitutions in the TM domains—may constrain the spatial orientation of α‐MSH within the binding pocket, facilitating the formation of additional hydrogen bonds and stabilizing the receptor–ligand complex. This finding establishes a direct link between MC1R structural variation, binding pocket architecture, and ligand‐binding affinity in *P. versicolor* .

The melanistic HSK population and the nearby non‐melanistic GZ population provide an ideal model for studying the ecological and evolutionary significance of phenotypic traits. Ecologically, substrate color matching is highly effective in reducing predation pressure through camouflage, a pattern that has been experimentally validated in *P. versicolor* (Tong et al. 2019; Wan et al. 2021). However, as ectothermic vertebrates, the potential association between such color variation and thermoregulation remains to be investigated, similar to patterns reported in *P. guinanensis* (Jin et al. 2016) and *P. putjatai* (Sun et al. 2024). On the other hand, color variation between the two populations has been shown to represent an adaptive trait rather than phenotypic plasticity (Tong et al. 2019). Given the absence of obvious geographical isolation between HSK and GZ, the melanistic population may have evolved under the joint effects of strong natural selection and ongoing gene flow. Although this study and our previous work have identified several genes associated with color variation, detailed genotype–phenotype mapping and genome‐wide analyses of gene flow based on high‐coverage genomic data are still needed to elucidate the genetic basis of color variation in *P. versicolor* . Furthermore, the number of samples with luminance values was limited; in particular, some genotypes were represented by only one or two individuals, resulting in a lack of representativeness. Therefore, we did not further investigate the relationship between genotype and phenotype within populations. This issue should be addressed in the future, as robust inferences of adaptive divergence typically require evidence of within‐population covariation, rather than relying exclusively on between‐population comparisons (Peiman and Robinson 2017; Yao and Fu 2025).

In summary, through molecular cloning and in silico protein structure analysis, we identified two amino acid substitutions (V165I and V237I) in the MC1R that are significantly associated with body color variation in *P. versicolor* . Our findings provide novel insights into how amino acid substitutions within TM domains modulate receptor–ligand‐binding affinity—specifically by reshaping the size of the MC1R‐binding pocket, which in turn enhances the interaction between MC1R and its ligand α‐MSH in melanistic individuals. To systematically elucidate the molecular mechanism underlying the extreme color variation in *P. versicolor* , future studies should focus on two key directions: first, investigating the effects of the V165I and V237I substitutions on MC1R expression levels and cellular localization; second, conducting functional experiments (such as in vitro real‐time binding efficiency assays) to validate the in silico predicted changes in binding ability. These follow‐up studies will complement the current findings and deepen our understanding of how MC1R mediates adaptive melanism in reptiles.

## Author Contributions

**Haojie Tong:** data curation (lead), formal analysis (lead), investigation (lead), methodology (lead), software (lead), writing – original draft (lead), writing – review and editing (lead). **Qianjin Lin:** formal analysis (equal), methodology (equal), software (equal), validation (equal), visualization (equal). **Shuangyue Shi:** formal analysis (equal), investigation (equal). **Kaixin Wang:** formal analysis (equal), investigation (equal). **Yaxin Guo:** formal analysis (equal), investigation (equal). **Richard P. Brown:** methodology (equal), project administration (equal), writing – review and editing (equal). **Yuanting Jin:** formal analysis (equal), funding acquisition (lead), project administration (lead), resources (lead), supervision (lead), writing – review and editing (equal).

## Funding

This work was supported by the National Natural Science Foundation of China, 32370441.

## Conflicts of Interest

The authors declare no conflicts of interest.

## Supporting information


**Data S1:** The nuclear variable loci and amino acid substitutions for each *P. versicolor* individual.

**Table S1:** The pigment‐related MC1R substitutions in vertebrata.

## Data Availability

Sequence data have been deposited in Figshare (https://figshare.com/s/f494669a50dc7149ca56).

## References

[ece373481-bib-0001] Barsh, G. , T. Gunn , L. He , S. Schlossman , and J. Duke‐Cohan . 2000. “Biochemical and Genetic Studies of Pigment‐Type Switching.” Pigment Cell Research 8: 48–53. 10.1034/j.1600-0749.13.s8.10.x 11041357

[ece373481-bib-0002] Buades, J. M. , V. Rodríguez , B. Terrasa , et al. 2013. “Variability of the *mc1r* Gene in Melanic and Non‐Melanic *Podarcis lilfordi* and *Podarcis pityusensis* From the Balearic Archipelago.” PLoS One 8: e53088. 23308144 10.1371/journal.pone.0053088 PMC3538740

[ece373481-bib-0003] Cavatao, F. G. , E. S. M. Pinto , M. J. Krause , C. S. Alho , and M. Dorn . 2024. “Molecular Basis of MC1R Activation: Mutation‐Induced Alterations in Structural Dynamics.” Proteins: Structure, Function, and Bioinformatics 92: 1297–1307. 10.1002/prot.26722 38923677

[ece373481-bib-0004] Chen, S. Y. , B. Zhu , C. Q. Yin , et al. 2017. “Palmitoylation‐Dependent Activation of MC1R Prevents Melanomagenesis.” Nature 549: 399–403. 28869973 10.1038/nature23887 PMC5902815

[ece373481-bib-0005] Chen, Y. , S. Tan , Q. W. Xu , et al. 2025. “Genomic Architecture Underlying the Striking Colour Variation in the Presence of Gene Flow for the Guinan Toad‐Headed Lizard.” Molecular Ecology 34: e17594. 39548709 10.1111/mec.17594

[ece373481-bib-0006] Corso, J. , G. L. Gonçalves , and T. R. O. de Freitas . 2012. “Sequence Variation in the Melanocortin‐1 Receptor (MC1R) Pigmentation Gene and Its Role in the Cryptic Coloration of Two South American Sand Lizards.” Genetics and Molecular Biology 35: 81–87. 22481878 10.1590/s1415-47572012005000015 PMC3313520

[ece373481-bib-0007] Cox, C. L. , A. R. D. Rabosky , and P. T. Chippindale . 2013. “Sequence Variation in the *Mc1r* Gene for a Group of Polymorphic Snakes.” Gene 513: 282–286. 23116942 10.1016/j.gene.2012.10.065

[ece373481-bib-0008] Feng, W. B. , Q. T. Zhou , C. Zheng , D. H. Yang , and M. W. Wang . 2025. “Structural Basis for the Constitutive Activity of the Melanocortin Receptor Family.” Structure 33: 1074–1087. 40157361 10.1016/j.str.2025.03.004

[ece373481-bib-0009] Forli, S. , R. Huey , M. E. Pique , M. F. Sanner , D. S. Goodsell , and A. J. Olson . 2016. “Computational Protein‐Ligand Docking and Virtual Drug Screening With the AutoDock Suite.” Nature Protocols 11: 905–919. 27077332 10.1038/nprot.2016.051 PMC4868550

[ece373481-bib-0010] Frandberg, P. A. , R. Muceniece , P. Prusis , J. Wikberg , and V. Chhajlani . 1994. “Evidence for Alternate Points of Attachment for Alpha‐MSH and Its Stereoisomer [Nle4, D‐Phe7]‐Alpha‐MSH at the Melanocortin‐1 Receptor.” Biochemical and Biophysical Research Communications 202: 1266–1271. 8060302 10.1006/bbrc.1994.2067

[ece373481-bib-0011] Garcia‐Borron, J. C. , B. L. Sanchez‐Laorden , and C. Jimenez‐Cervantes . 2005. “Melanocortin‐1 Receptor Structure and Functional Regulation.” Pigment Cell Research 18: 393–410. 16280005 10.1111/j.1600-0749.2005.00278.x

[ece373481-bib-0012] Garcia‐Elfring, A. , H. L. Roffey , J. M. Abergas , et al. 2025. “A Ball Python Colour Morph Implicates *MC1R* in Melanophore‐Xanthophore Distribution and Pattern Formation.” Pigment Cell & Melanoma Research 38: e13215. 39609249 10.1111/pcmr.13215

[ece373481-bib-0013] Guernsey, M. W. , L. Ritscher , M. A. Miller , D. A. Smith , T. Schöneberg , and M. D. Shapiro . 2013. “A Val85Met Mutation in Melanocortin‐1 Receptor Is Associated With Reductions in Eumelanic Pigmentation and Cell Surface Expression in Domestic Rock Pigeons ( *Columba livia* ).” PLoS One 8: e74475. 23977400 10.1371/journal.pone.0074475 PMC3744500

[ece373481-bib-0014] Hassan, N. M. , A. A. Alhossary , Y. G. Mu , and C. K. Kwoh . 2017. “Protein‐Ligand Blind Docking Using QuickVina‐W With Inter‐Process Spatio‐Temporal Integration.” Scientific Reports 7: 15451. 29133831 10.1038/s41598-017-15571-7 PMC5684369

[ece373481-bib-0015] Hauser, M. , H. Signer‐Hasler , L. Küttel , et al. 2022. “Identification of Two New Recessive *MC1R* Alleles in Red‐Coloured Evolener Cattle and Other Breeds.” Animal Genetics 53: 427–435. 35451516 10.1111/age.13206 PMC9373916

[ece373481-bib-0016] Horrell, E. M. W. , M. C. Boulanger , and J. A. D'Orazio . 2016. “Melanocortin 1 Receptor: Structure, Function, and Regulation.” Frontiers in Genetics 7: 95. 27303435 10.3389/fgene.2016.00095 PMC4885833

[ece373481-bib-0017] Jin, Y. T. , D. Aguilar‐Gómez , D. Y. C. Brandt , et al. 2022. “Population Genomics of Variegated Toad‐Headed Lizard *Phrynocephalus versicolor* and Its Adaptation to the Colorful Sand of the Gobi Desert.” Genome Biology and Evolution 14: evac076. 35679302 10.1093/gbe/evac076 PMC9260186

[ece373481-bib-0018] Jin, Y. T. , H. J. Tong , G. Shao , et al. 2020. “Dorsal Pigmentation and Its Association With Functional Variation in MC1R in a Lizard From Different Elevations on the Qinghai‐Tibetan Plateau.” Genome Biology and Evolution 12: 2303–2313. 33095228 10.1093/gbe/evaa225 PMC7719228

[ece373481-bib-0052] Jin, Y. T. , H. J. Tong , and K. L. Zhang . 2016. “The Impact of Phenotypic Characteristics on Thermoregulation in a Cold‐Climate Agamid Lizard, *Phrynocephalus guinanensis* .” Asian Herpetological Research 7, no. 3: 210–219.

[ece373481-bib-0019] Kawaguchi, F. , A. Shaku , M. K. Shah , J. S. Masangkay , H. Mannen , and S. Sasazaki . 2025. “Detection of MC1R Genetic Variants and Their Association With Coat Color in Asian Goats.” Animals 15: 2026. 40723488 10.3390/ani15142026 PMC12291834

[ece373481-bib-0020] Laurent, S. , S. P. Pfeifer , M. L. Settles , et al. 2016. “The Population Genomics of Rapid Adaptation: Disentangling Signatures of Selection and Demography in White Sands Lizards.” Molecular Ecology 25: 306–323. 26363411 10.1111/mec.13385

[ece373481-bib-0021] Ma, S. S. , Y. Chen , A. T. Dai , et al. 2021. “Structural Mechanism of Calcium‐Mediated Hormone Recognition and Gβ Interaction by the Human Melanocortin‐1 Receptor.” Cell Research 31: 1061–1071. 34453129 10.1038/s41422-021-00557-y PMC8486761

[ece373481-bib-0022] Matsuba, C. 2012. “Geographic Variations of Melanocortine 1 Receptor Gene (*MC1R*) in the Common Frog ( *Rana temporaria* ) in Northern Europe.” Amphibia‐Reptilia 33: 105–111.

[ece373481-bib-0023] Metallinou, M. , J. Cervenka , P. A. Crochet , et al. 2015. “Species on the Rocks: Systematics and Biogeography of the Rock‐Dwelling *Ptyodactylus* Geckos (Squamata: Phyllodactylidae) in North Africa and Arabia.” Molecular Phylogenetics and Evolution 85: 208–220. 25724867 10.1016/j.ympev.2015.02.010

[ece373481-bib-0024] Nachman, M. W. , H. E. Hoekstra , and S. L. D'Agostino . 2003. “The Genetic Basis of Adaptive Melanism in Pocket Mice.” Proceedings of the National Academy of Sciences of the United States of America 100: 5268–5273. 12704245 10.1073/pnas.0431157100 PMC154334

[ece373481-bib-0025] Nunes, V. L. , A. Miraldo , M. A. Beaumont , R. K. Butlin , and O. S. Paulo . 2011. “Association of MC1R Variants With Ecologically Relevant Phenotypes in the European Ocellated Lizard, *Lacerta lepida* .” Journal of Evolutionary Biology 24: 2289–2298. 21812853 10.1111/j.1420-9101.2011.02359.x

[ece373481-bib-0026] Peiman, K. S. , and B. W. Robinson . 2017. “Comparative Analyses of Phenotypic Trait Covariation Within and Among Populations.” American Naturalist 190: 451–468. 10.1086/693482 28937814

[ece373481-bib-0027] Qi, Y. , X. Zhang , Y. Pang , B. Yuan , and J. Cheng . 2023. “Identification of Polymorphism in the *MC1R* Gene and Its Association With the Melanin Content in Feathers of Chinese Yellow Quails.” Brazilian Journal of Poultry Science 25: eRBCA20221648.

[ece373481-bib-0028] Ribeiro, M. A., Jr. , K. Tamar , E. Maza , et al. 2022. “Taxonomic Revision of the *Tropiocolotes nattereri* (Squamata, Gekkonidae) Species Complex, With the Description of a New Species From Israel, Jordan and Saudi Arabia.” Zoologica Scripta 51: 288–309.

[ece373481-bib-0029] Richardson, J. , P. R. Lundegaard , N. L. Reynolds , et al. 2008. “mc1r Pathway Regulation of Zebrafish Melanosome Dispersion.” Zebrafish 5: 289–295. 19133827 10.1089/zeb.2008.0541

[ece373481-bib-0030] Ringholm, A. , J. Klovins , R. Rudzish , S. Phillips , and H. B. Schith . 2004. “Pharmacological Characterization of Loss of Function Mutations of the Human Melanocortin 1 Receptor That Are Associated With Red Hair.” Journal of Investigative Dermatology 123: 917–923. 15482480 10.1111/j.0022-202X.2004.23444.x

[ece373481-bib-0031] Rosenblum, E. B. , H. E. Hoekstra , and M. W. Nachman . 2004. “Adaptive Reptile Color Variation and the Evolution of the *Mclr* Gene.” Evolution 58: 1794–1808. 15446431 10.1111/j.0014-3820.2004.tb00462.x

[ece373481-bib-0032] Rosenblum, E. B. , H. Römpler , T. Schöneberg , and H. E. Hoekstra . 2010. “Molecular and Functional Basis of Phenotypic Convergence in White Lizards at White Sands.” Proceedings of the National Academy of Sciences of the United States of America 107: 2113–2117. 20080544 10.1073/pnas.0911042107 PMC2836677

[ece373481-bib-0033] Rozas, J. , A. Ferrer‐Mata , J. C. Sánchez‐DelBarrio , et al. 2017. “DnaSP 6: DNA Sequence Polymorphism Analysis of Large Data Sets.” Molecular Biology and Evolution 34: 3299–3302. 29029172 10.1093/molbev/msx248

[ece373481-bib-0034] Schrödinger, L. , and W. DeLano . 2020. “PyMOL.” http://www.pymol.org/pymol.

[ece373481-bib-0035] Schwochow, D. , S. Bornelöv , T. X. Jiang , et al. 2021. “The Feather Pattern Autosomal Barring in Chicken Is Strongly Associated With Segregation at the *MC1R* Locus.” Pigment Cell & Melanoma Research 34: 1015–1028. 33793042 10.1111/pcmr.12975 PMC8484376

[ece373481-bib-0036] Song, F. B. , L. Wang , Z. H. Yang , et al. 2022. “Transcriptome Analysis Reveals the Complex Regulatory Pathway of Background Color in Juvenile *Plectropomus leopardus* Skin Color Variation.” International Journal of Molecular Sciences 23: 11186. 36232493 10.3390/ijms231911186 PMC9569894

[ece373481-bib-0037] Sun, B. J. , W. M. Li , P. Lv , et al. 2024. “Genetically Encoded Lizard Color Divergence for Camouflage and Thermoregulation.” Molecular Biology and Evolution 41: msae009. 38243850 10.1093/molbev/msae009 PMC10835340

[ece373481-bib-0038] Tamura, K. , G. Stecher , D. Peterson , A. Filipski , and S. Kumar . 2013. “MEGA6: Molecular Evolutionary Genetics Analysis Version 6.0.” Molecular Biology and Evolution 30: 2725–2729. 24132122 10.1093/molbev/mst197 PMC3840312

[ece373481-bib-0039] Tao, X. Q. , Z. G. Jiang , S. N. Ji , H. J. Chu , D. D. Yang , and C. Li . 2018. “Influence of Light Intensity and Substrate Color on Dorsal Gray Color Change in *Phrynocephalus helioscopus* and *Phrynocephalus grumgrzimailoi* .” Journal of Arid Environments 157: 22–26.

[ece373481-bib-0040] Tong, H. J. , J. S. Li , Y. B. Wo , et al. 2019. “Effects of Substrate Color on Intraspecific Body Color Variation in the Toad‐Headed Lizard, *Phrynocephalus versicolor* .” Ecology and Evolution 9: 10253–10262. 31624549 10.1002/ece3.5545 PMC6787858

[ece373481-bib-0041] Tong, H. J. , G. Shao , L. J. Wang , et al. 2023. “Association of a Single Amino Acid Replacement With Dorsal Pigmentation in a Lizard From the Qinghai‐Tibetan Plateau.” International Journal of Biological Macromolecules 242: 124907. 37230451 10.1016/j.ijbiomac.2023.124907

[ece373481-bib-0042] Tong, H. J. , K. L. Zhang , Y. H. Liu , L. X. Zhang , W. Zhao , and J. Yuanting . 2016. “Effects of Substrate Color on the Body Color Variation of Two Agamid Lizards, *Phrynocephalus versicolor* and *P. frontalis* .” Biodiversity Science 24: 1039–1044 (in Chinese with English abstract).

[ece373481-bib-0043] Valverde, P. , E. Healy , I. Jackson , J. L. Rees , and A. J. Thody . 1995. “Variants of the Melanocyte‐Stimulating Hormone Receptor Gene Are Associated With Red Hair and Fair Skin in Humans.” Nature Genetics 11: 328–330. 7581459 10.1038/ng1195-328

[ece373481-bib-0051] Wan, L. X. , Z. X. Liu , T. Wang , et al. 2021. “Camouflage Versus Running Performance as Strategies Against Predation in a Lizard Inhabiting Different Habitats.” Ecology and Evolution 11: 17409–17416. 34938517 10.1002/ece3.8374 PMC8668757

[ece373481-bib-0044] Wang, L. M. , M. K. Luo , H. R. Yin , W. B. Zhu , J. J. Fu , and Z. J. Dong . 2020. “Effects of Background Adaptation on the Skin Color of Malaysian Red Tilapia.” Aquaculture 521: 735061.

[ece373481-bib-0045] Wang, Y. Z. , and J. Z. Fu . 2004. “Cladogenesis and Vicariance Patterns in the Toad‐Headed Lizard *Phrynocephalus versicolor* Species Complex.” Copeia 2004: 199–206.

[ece373481-bib-0046] Xiong, Q. , J. Chai , M. X. Chen , and Y. X. Tao . 2016. “Identification and Pharmacological Analyses of Eight Naturally Occurring Caprine Melanocortin‐1 Receptor Mutations in Three Different Goat Breeds.” General and Comparative Endocrinology 235: 1–10. 27229376 10.1016/j.ygcen.2016.05.023

[ece373481-bib-0047] Yang, Y. K. , V. K. Mishra , M. Chen , E. Duffee , R. Dimmitt , and C. M. Harmon . 2013. “Molecular Characterization of Human Melanocortin‐5 Receptor Ligand‐Receptor Interaction.” Biochemistry 52: 1737–1745. 23414113 10.1021/bi3013593

[ece373481-bib-0048] Yao, Z. Y. , and J. Z. Fu . 2025. “Optic Tectum Size Correlates Positively With Eye Size Across, but Not Within, Populations of Asiatic Toads Along Altitudinal Gradients.” Biology Letters 21: 20250487. 41159215 10.1098/rsbl.2025.0487 PMC12569636

[ece373481-bib-0049] Zheng, W. , Q. Wuyun , Y. Li , et al. 2025. “Deep‐Learning‐Based Single‐Domain and Multidomain Protein Structure Prediction With D‐I‐TASSER.” Nature Biotechnology. In press. 10.1038/s41587-025-02654-4. PMC13090124 40410405

