# Supplementary material for: Associations of MC1R Substitutions With Body Color Variation in a Desert Lizard
Source: Ecol Evol. 2026 Apr 23;16(4):e73481. doi: 10.1002/ece3.73481 (PMC13106876; doi:10.1002/ece3.73481)
Supplement: Supplementary file 2 — Table S1: The pigment‐related MC1R substitutions in vertebrata. [file ECE3-16-e73481-s001.docx]

**Supplementary Table 1** The pigment related MC1R substitutions in vertebrata

| Class | Protein region | Amino acid substitutions | Species | References |
| --- | --- | --- | --- | --- |
| Mammalia | N-terminus | R18S | *Chaetodipus intermedius* | Nachman et al. 2003 |
|  |  | C33F | *Mus musculus* | Suzuki et al. 2020 |
|  |  | R34W | *Homo sapiens* | Hu et al. 2014 |
|  |  | C35Y | *Homo sapiens* | Fargnoli et al. 2003 |
|  | TM1 | V38M | *Homo sapiens* | Fargnoli et al. 2003 |
|  |  | S41C | *Homo sapiens* | Hu et al. 2014 |
|  |  | F45L | *Homo sapiens* | Pastorino et al. 2004 |
|  |  | L48P | *Dama dama* | Reissmann et al. 2024 |
|  |  | V60L | *Homo sapiens* | Box et al. 1997 |
|  | IL1 | R67W | *Homo sapiens* | Branicki et al. 2007 |
|  |  | R67Q | *Homo sapiens* | Rana et al. 1999 |
|  |  | L69V | *Homo sapiens* | Hu et al. 2014 |
|  |  | S71L | *Mus musculus* | Robbins et al. 1993 |
|  | TM2 | M73K | *Ovis aries* | Våge et al. 1999 |
|  |  | A81P | *Homo sapiens* | Bastiaens et al. 2001 |
|  |  | A81V | *Capra hircus* | Fontanesi et al. 2009 |
|  |  | S83P | *Equus caballus*, *Homo sapiens* | Marklund et al. 1996; John et al. 2003 |
|  |  | D84E | *Homo sapiens* | Valverde et al. 1995 |
|  |  | V92M | *Homo sapiens* | Valverde et al. 1995 |
|  |  | E94K | *Mus musculus* | Robbins et al. 1993 |
|  |  | T95M | *Homo sapiens* | Valverde et al. 1995 |
|  |  | L99P | Cattle, *Sus scrofa*, *Homo sapiens* | Klungland et al. 1995; Kijas et al. 1998; Hu et al. 2014 |
|  |  | L100P | *Mus musculus* | Robbins et al. 1993 |
|  | EL1 | G104S | *Homo sapiens*, *Bubalus bubalis* | Bastiaens et al. 2001; Miao et al. 2010 |
|  | TM3 | D117V | *Homo sapiens* | Hu et al. 2014 |
|  |  | I120T | *Homo sapiens* | Fargnoli et al. 2003; Kawaguchi et al. 2025 |
|  |  | D121N | *Sus scrofa*, *Ovis aries* | Kijas et al. 1998; Våge et al. 1999 |
|  |  | V122M | *Homo sapiens* | Jimenez-Cervantes et al. 2001 |
|  |  | C123R | *Vulpes vulpes* | Våge et al. 1997 |
|  |  | S130F | *Homo sapiens* | Hu et al. 2014 |
|  |  | A139T | *Homo sapiens* | Scherer et al. 2009 |
|  |  | V140M | *Homo sapiens* | Hu et al. 2014 |
|  |  | V140G | *Homo sapiens* | Hu et al. 2014 |
|  |  | R142C | *Homo sapiens* | Matichard et al. 2004 |
|  |  | R142H | *Homo sapiens* | Box et al. 1997 |
|  | IL2 | R151C | *Homo sapiens* | Box et al. 1997 |
|  |  | Y152X | *Homo sapiens* | John et al. 2002 |
|  |  | I155T | *Homo sapiens* | Box et al. 1997 |
|  |  | V156A | *Homo sapiens* | Scherer et al. 2009 |
|  |  | R160W | *Homo sapiens* | Box et al. 1997 |
|  |  | R160Q | *Homo sapiens* | Pastorino et al. 2004 |
|  | TM4 | R163Q | *Homo sapiens* | Box et al. 1997 |
|  |  | A171D | *Homo sapiens* | John et al. 2002 |
|  |  | H183Q | *Mus musculus* | Robbins et al. 1993 |
|  | TM5 | V205L | *Homo sapiens* | Hu et al. 2014 |
|  |  | R213W | *Homo sapiens* | Pastorino et al. 2004 |
|  |  | A218T | *Homo sapiens* | Landi et al. 2005 |
|  | IL3 | A222S | *Homo sapiens* | Hu et al. 2014 |
|  |  | R223W | *Homo sapiens* | Hu et al. 2014 |
|  |  | Q225X | *Capra hircus* | Fontanesi et al. 2009 |
|  |  | K226E | *Homo sapiens* | Kawaguchi et al. 2025 |
|  |  | G236D | *Homo sapiens*, *Dama dama* | Hu et al. 2014; Reissmann et al. 2024 |
|  | TM6 | A240T | *Sus scrofa* | Kijas et al. 1998 |
|  |  | F250V | *Capra hircus* | Fontanesi et al. 2009; Kawaguchi et al. 2025 |
|  |  | P256S | *Homo sapiens* | John et al. 2002 |
|  | EL3 | C267X | *Homo sapiens* | Hu et al. 2014 |
|  |  | C267W | *Capra hircus* | Fontanesi et al. 2009 |
|  |  | G274S | *Homo sapiens* | Scherer et al. 2008 |
|  | TM7 | K278E | *Homo sapiens* | Bastiaens et al. 2001 |
|  |  | N281S | *Homo sapiens* | Liboutet et al. 2006 |
|  |  | A285T | *Homo sapiens* | Hu et al. 2014 |
|  |  | A285G | *Homo sapiens* | Hu et al. 2014 |
|  |  | I287M | *Homo sapiens* | Harding et al. 2000 |
|  |  | C289R | *Homo sapiens*, | Perez-Oliva et al. 2009 |
|  |  | D294H | *Homo sapiens* | Valverde et al. 1995 |
|  |  | Y298H | *Homo sapiens* | Matichard et al. 2004 |
|  |  | Y298C | *Ursus americanus* | Ritland et al. 2001 |
|  |  | A299T | *Homo sapiens* | Box et al. 1997 |
|  | C-terminus | R306H | *Homo sapiens* | Liboutet et al. 2006 |
|  |  | K310Q | *Homo sapiens* | Hu et al. 2014 |
|  |  | W317C | *Homo sapiens* | Hu et al. 2014 |
| Aves | IL1 | S69L | *Columba livia* | Guernsey et al. 2013 |
|  | TM2 | V85M | *Columba livia* | Guernsey et al. 2013 |
|  |  | E94K | *Coereba flaveola*, | Theron et al. 2001 |
|  | TM3 | D119N | *Gallus gallus* | Horecka et al. 2024 |
|  |  | M120I | *Coturnix japonica* | Qi et al. 2023 |
|  |  | V128I | *Falco rusticolus* | Johnson et al. 2012 |
|  |  | L133Q | *Gallus gallus* | Schwochow et al. 2021 |
| Reptilia | N-terminus | T20P | *P. theobaldi* | Jin et al. 2020 |
|  |  | V22M | *P. theobaldi* | Jin et al. 2020 |
|  |  | R28Q | *P. theobaldi* | Jin et al. 2020 |
|  | TM1 | S50N | *P. regius* | Garcia-Elfrin et al. 2025 |
|  |  | V52M | *P. theobaldi* | Jin et al. 2020 |
|  | TM4 | V165I | *P. theobaldi*, *P. versicolor* | Jin et al. 2020; In this study |
|  |  | V168I | *H. maculata* | Rosenblum et al., 2004; 2010 |
|  |  | T170I | *A. inornate* | Rosenblum et al., 2004; 2010 |
|  |  | S172C | *L. lepida lepida* | Nunes et al. 2011 |
|  |  | E183K | *P. erythrurus* | Tong et al. 2023 |
|  | TM5 | H208Y | *S. undulatus* | Rosenblum et al., 2004; 2010 |
|  | TM6 | V237I | *P. versicolor* | In this study |
| Fish | TM3 | G120S | *Astroblepus pholeter* | Espinasa et al. 2018 |
|  | TM6 | L251M | *Astroblepus pholeter* | Espinasa et al. 2018 |

References:

Bastiaens, M., ter Huurne, J., Gruis, N., Bergman, W., Westendorp, R., et al. 2001. The melanocortin-1-receptor gene is the major freckle gene. *Human Molecular Genetics* **10**: 1701–1708.

Box, N. F., Wyeth, J. R., O’Gorman, L. E., Martin, N. G., and Sturm, R. A. 1997. Characterization of melanocyte stimulating hormone receptor variant alleles in twins with red hair. *Human Molecular Genetics* **6**: 1891–1897.

Branicki, W., Brudnik, U., Kupiec, T., Wolañska‐Nowak, P., and Wojas‐Pelc, A. 2007. Determination of phenotype associated SNPs in the *MC1R* gene. *Journal of Forensic Sciences* **52**: 349–354.

Espinasa, L., Robinson, J., and Espinasa, M. 2018. *Mc1r* gene in *Astroblepus pholeter* and *Astyanax mexicanus*: convergent regressive evolution of pigmentation across cavefish species. *Developmental Biology* **441**: 305–310.

Fargnoli, M. C., Chimenti, S., Keller, G., Höfler, H., and Peris, K. 2003. Identification of four novel melanocortin 1 receptor (*MC1R*) gene variants in a Mediterranean population. *Human Mutation* **21**: 655–655.

Fontanesi, L., Beretti, F., Riggio, V., Gómez González, E., Dall’Olio, S., et al. 2009. Copy number variation and missense mutations of the agouti signaling protein (*ASIP*) gene in goat breeds with different coat colors. *Cytogenetic and Genome Research* **126**: 333–347.

Garcia‐Elfring, A., Roffey, H. L., Abergas, J. M., Wuyts, J., Hendry, A. P., et al. 2025. A ball python colour morph implicates *MC1R* in melanophore–xanthophore distribution and pattern formation. *Pigment Cell and Melanoma Research* **38**: e13215.

Guernsey, M. W., Ritscher, L., Miller, M. A., Smith, D. A., Schöneberg, T., et al. 2013. A Val85Met mutation in melanocortin-1 receptor is associated with reductions in eumelanic pigmentation and cell surface expression in domestic rock pigeons (*Columba livia*). *PLoS One* **8**: e74475.

Harding, R. M., Healy, E., Ray, A. J., Ellis, N. S., Flanagan, N., et al. 2000. Evidence for variable selective pressures at *MC1R*. *The American Journal of Human Genetics* **66**:1351–1361.

Horecka, B., Wojciechowski, W., Drabik, K., Wengerska, K., and Batkowska, J. 2024. Characterization of the coding sequence of the *MC1R* (melanocortin 1 receptor) gene of *Ayam Cemani* black chickens. *Animals* **14**: 2507.

Hu, H.-H., Benfodda, M., Dumaz, N., Gazal, S., Descamps, V., et al. 2014. A large French case-control study emphasizes the role of rare *MC1R* variants in melanoma risk. *BioMed Research International* **2014**: 1–10.

Jiménez-Cervantes, C., Germer, S., González, P., Sánchez, J., Sánchez, C. O., et al. 2001. Thr40 and Met122 are new partial loss-of-function natural mutations of the human melanocortin 1 receptor. *FEBS Letters* **508**: 44–48.

Jin, Y., Tong, H., Shao, G., Li, J., Lv, Y., et al. 2020. Dorsal pigmentation and its association with functional variation in *MC1R* in a lizard from different elevations on the Qinghai–Tibetan Plateau. *Genome Biology and Evolution* **12**: 2303–2313.

John, P. R., Makova, K., Li, W. H., Jenkins, T., and Ramsay, M. 2003. DNA polymorphism and selection at the melanocortin‐1 receptor gene in normally pigmented Southern African individuals. *Annals of the New York Academy of Sciences* **994**: 299–306.

John, P. R., and Ramsay, M. 2002. Four novel variants in *MC1R* in red-haired South African individuals of European descent: S83P, Y152X, A171D, P256S. *Human Mutation* **19**: 461–462.

Johnson, J. A., Ambers, A. D., and Burnham, K. K. 2012. Genetics of plumage color in the Gyrfalcon (*Falco rusticolus*): Analysis of the melanocortin-1 receptor gene. *Journal of Heredity* **103**: 315–321.

Kawaguchi, F., Shaku, A., Shah, M. K., Masangkay, J. S., Mannen, H., et al. 2025. Detection of *MC1R* genetic variants and their association with coat color in Asian goats. *Animals* **15**: 2026.

Kijas, J. M. H., Wales, R., Törnsten, A., Chardon, P., Moller, M., et al. 1998. Melanocortin receptor 1 (*MC1R*) mutations and coat color in pigs. *Genetics* **150**: 1177–1185.

Klungland, H., Vage, D. I., Gomez-Raya, L., Adalsteinsson, S., and Lien, S. 1995. The role of melanocyte-stimulating hormone (*MSH*) receptor in bovine coat color determination. *Mammalian Genome* **6**: 636–639.

Landi, M. T., Kanetsky, P. A., Tsang, S., Gold, B., Munroe, D., et al. 2005. *MC1R*, *ASIP*, and DNA repair in sporadic and familial melanoma in a Mediterranean population. *Journal of the National Cancer Institute* **97**: 998–1007.

Liboutet, M., Portela, M., Delestaing, G., Vilmer, C., Dupin, N., et al. 2006. *MC1R* and *PTCH* gene polymorphism in French patients with basal cell carcinomas. *Journal of Investigative Dermatology* **126**: 1510–1517.

Marklund, L., Moller, M. J., Sandberg, K., and Andersson, L. 1996. A missense mutation in the gene for melanocyte-stimulating hormone receptor (*MC1R*) is associated with the chestnut coat color in horses. *Mammalian Genome* **7**: 895–899.

Matichard, E., Verpillat, P., Meziani, R., Gérard, B., Descamps, V., et al. 2004. Melanocortin 1 receptor (*MC1R*) gene variants may increase the risk of melanoma in France independently of clinical risk factors and UV exposure. *Journal of Medical Genetics* **41**: e13–e13.

Miao, Y., Wu, G., Wang, L., Li, D., Tang, S., et al. 2010. The role of *MC1R* gene in buffalo coat color. *Science China Life Sciences* **53**: 267–272.

Nachman, M. W., Hoekstra, H. E., and D’Agostino, S. L. 2003. The genetic basis of adaptive melanism in pocket mice. *Proceedings of the National Academy of Sciences* **100**: 5268–5273.

Nunes, V. L., Miraldo, A., Beaumont, M. A., Butlin, R. K., and Paulo, O. S. 2011. Association of *Mc1r* variants with ecologically relevant phenotypes in the European ocellated lizard, *Lacerta lepida*. *Journal of Evolutionary Biology* **24**: 2289–2298.

Pastorino, L., Cusano, R., Bruno, W., Lantieri, F., Origone, P., et al. 2004. Novel *MC1R* variants in Ligurian melanoma patients and controls. *Human Mutation* **24**: 103–103.

Pérez Oliva, A. B., Fernéndez, L. P., DeTorre, C., Herráiz, C., Martínez-Escribano, J. A., et al. 2009. Identification and functional analysis of novel variants of the human melanocortin 1 receptor found in melanoma patients. *Human Mutation* **30**: 811–822.

Qi, Y., Zhang, X., Pang, Y., Yuan, B., and Cheng, J. 2023. Identification of polymorphism in the *MC1R* gene and its association with the melanin content in feathers of Chinese yellow quails. *Brazilian Journal of Poultry Science* **25**: eRBCA-2022.

Rana, B. K., Hewett-Emmett, D., Jin, L., Chang, B. H., Sambuughin, N., et al. 1999. High polymorphism at the human melanocortin 1 receptor locus. *Genetics* **151**: 1547–1557.

Reissmann, M., Ullrich, E., Bergfeld, U., and Ludwig, A. 2024. Agouti-signaling protein and melanocortin-1-receptor mutations associated with coat color phenotypes in fallow deer (*Dama dama*). *Genes* **15**: 1055.

Ritland, K., Newton, C., and Marshall, H. D. 2001. Inheritance and population structure of the white-phased “Kermode” black bear. *Current Biology* **11**: 1468–1472.

Robbins, L. S., Nadeau, J. H., Johnson, K. R., Kelly, M. A., Roselli-Rehfuss, L., et al. 1993. Pigmentation phenotypes of variant extension locus alleles result from point mutations that alter MSH receptor function. *Cell* **72**: 827–834.

Rosenblum, E. B., Hoekstra, H. E., and Nachman, M. W. 2004. Adaptive reptile color variation and the evolution of the *MC1R* gene. *Evolution* **58**: 1794–1808.

Rosenblum, E. B., Römpler, H., Schöneberg, T., and Hoekstra, H. E. 2010. Molecular and functional basis of phenotypic convergence in white lizards at White Sands. *Proceedings of the National Academy of Sciences* **107**: 2113–2117.

Scherer, D., Bermejo, J. L., Rudnai, P., Gurzau, E., Koppova, K., et al. 2008. *MC1R* variants associated susceptibility to basal cell carcinoma of skin: interaction with host factors and *XRCC3* polymorphism. *International Journal of Cancer* **122**: 1787–1793.

Scherer, D., Nagore, E., Bermejo, J. L., Figl, A., Botella‐Estrada, R., et al. 2009. Melanocortin receptor 1 variants and melanoma risk: a study of 2 European populations. *International Journal of Cancer* **125**: 1868–1875.

Schwochow, D., Bornelöv, S., Jiang, T., Li, J., Gourichon, D., et al. 2021. The feather pattern *autosomal barring* in chicken is strongly associated with segregation at the *MC1R* locus. *Pigment Cell and Melanoma Research* **34**: 1015–1028.

Suzuki, H., Kinoshita, G., Tsunoi, T., Noju, K., and Araki, K. 2020. Mouse hair significantly lightened through replacement of the cysteine residue in the N-terminal domain of Mc1r using the CRISPR/Cas9 system. *Journal of Heredity* **111**: 640–645.

Theron, E., Hawkins, K., Bermingham, E., Ricklefs, R. E., and Mundy, N. I. 2001. The molecular basis of an avian plumage polymorphism in the wild: a melanocortin-1-receptor point mutation is perfectly associated with the melanic plumage morph of the bananaquit, *Coereba flaveola*. *Current Biology* **11**: 550–557.

Tong, H., Shao, G., Wang, L., Li, J., Wang, T., et al. 2023. Association of a single amino acid replacement with dorsal pigmentation in a lizard from the Qinghai-Tibetan Plateau. *International Journal of Biological Macromolecules* **242**: 124907.

Våge, D. I., Klungland, H., Lu, D., and Cone, R. D. 1999. Molecular and pharmacological characterization of dominant black coat color in sheep. *Mammalian Genome* **10**: 39–43.

Våge, D. I., Lu, D., Klungland, H., Lien, S., Adalsteinsson, S., et al. 1997. A non-epistatic interaction of *agouti* and *extension* in the fox, *Vulpes vulpes*. *Nature Genetics* **15**: 311–315.

Valverde, P., Healy, E., Jackson, I., Rees, J. L., and Thody, A. J. 1995. Variants of the melanocyte–stimulating hormone receptor gene are associated with red hair and fair skin in humans. *Nature Genetics* **11**: 328–330.
